# The Role of NETosis in Systemic Lupus Erythematosus

**DOI:** 10.33696/immunology.1.008

**Published:** 2019-11-12

**Authors:** Ryan Salemme, Lauren N. Peralta, Sri Harika Meka, Nivetha Pushpanathan, Jessy J Alexander

**Affiliations:** Section of Nephrology, Department of Medicine, SUNY at Buffalo, Buffalo, NY 14203, USA

**Keywords:** Systemic lupus erythematosus, Netosis

## Abstract

Systemic lupus erythematosus is an autoimmune disease affecting multiple organs with devastating pathological consequences. Current treatment regimens largely rely on immunosuppressants and corticosteroids to attenuate autoimmune activity. However, such treatments have toxic side effects, often lacks efficacy, and inherently leaves the patient prone to infections, making the discovery of novel biomarkers and therapeutic targets an urgent need. Neutrophil extracellular traps (NETs) that participate in host defense are generated by neutrophils by a process called NETosis. NETs play an important role in the pathogenesis of SLE. In this review, we discuss the current literature regarding the role of NETs in SLE while entertaining the possibility that NETosis could serve as therapeutic targets thereby rendering the treatment more specific and effective in comparison to the current lupus therapy.

## Background

Systemic Lupus Erythematosus (SLE) is a devastating autoimmune disease that affects women to men at a ratio of 9:1 and is predominant in those of African ancestry. In SLE, the presence of autoantigens results in aberrant immune activation leading to systemic inflammation that predominantly affects the brain, kidneys, blood, and skin. Current guidelines recommend treatment with immunosuppressive drugs like prednisone, cyclophosphamide, azathioprine, and even some antimalarial drugs [1,2]. However, such drugs have limited efficacy, and result in toxic side effects. Therefore, research into the mechanism of the pathogenesis of SLE in order to identify novel therapies is important.

Autoantibodies generated in SLE induce influx of neutrophils to different sites of the body. Neutrophils are the body’s first line of defense against pathogens and are the most abundant leukocytes in the blood stream. The normal percentage of neutrophils (50%) can increase to 80% on site of a bacterial infection and detection of tissue inflammation. Neutrophils utilize various mechanisms of defense including phagocytosis, nicotinamide adenine dinucleotide phosphate (NADPH) oxidative burst, as well as the release of reactive oxidative species (ROS) and enzymes. More recently, it was discovered that dying neutrophils release ‘spider web’ like chromatin fibers called neutrophil extracellular traps (NETs). The release of NETs by activated neutrophils is known as NETosis [3]. These NETs are constructed of decondensed chromatin, histones, granules, and components with bactericidal activity. Antimicrobial enzymes such as myeloperoxidase (MPO), neutrophil elastase (NE), cathelicidins like LL-37, histones, proteinase 3, cathepsin, lactoferrin, or gelatinase are expelled with NETs, enabling them to entrap, inhibit, and kill invading pathogens in an extracellular manner, rather than the canonical method of phagocytosis [3,4].

**Figure 1:**
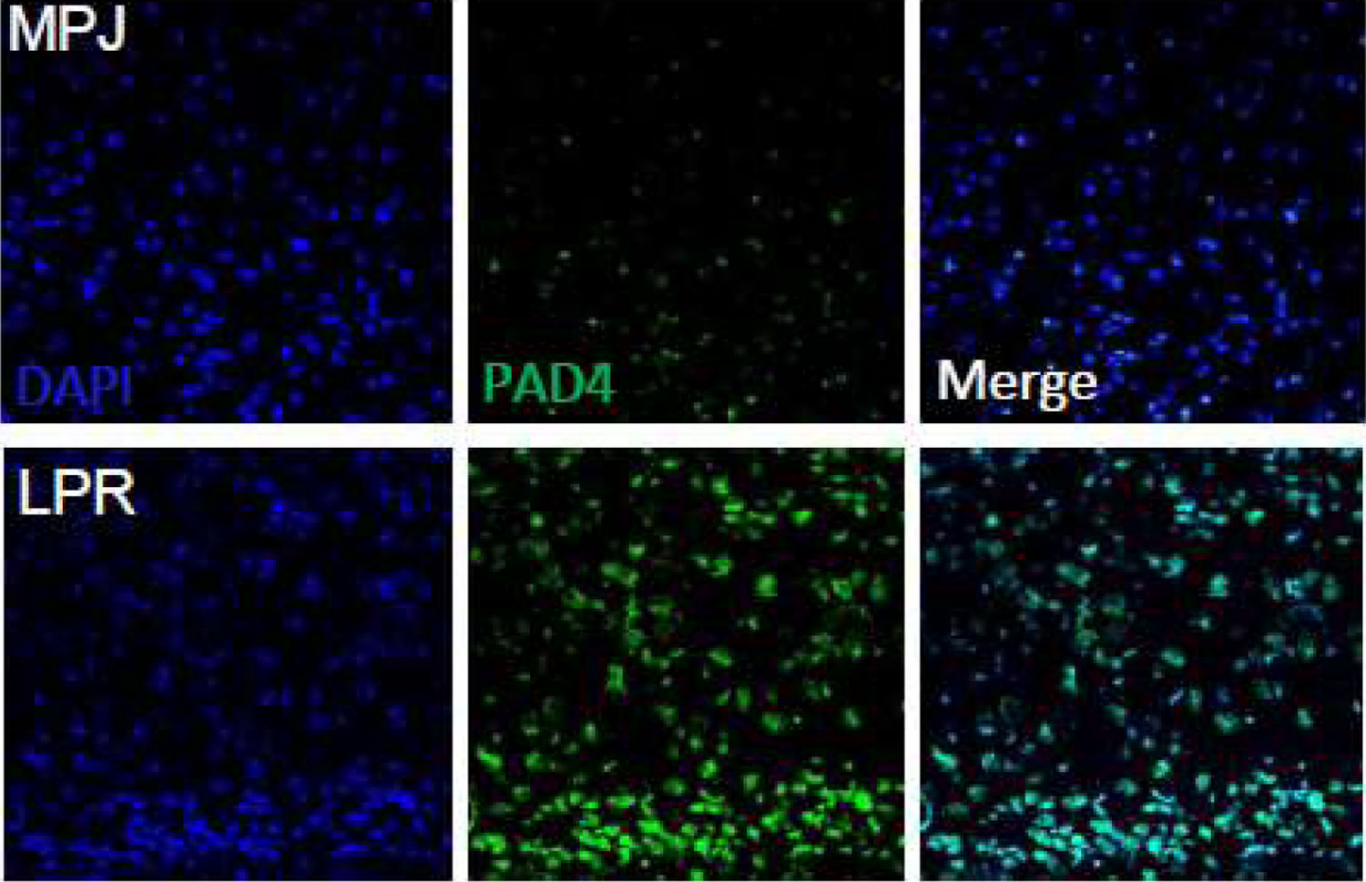
PAD_4_ expression is increased in experimental lupus brain. 8 μm frozen brain cortical sections obtained from MRL/MPJ congenic controls and MRL/*lpr* lupus mice were air dried, fixed in 4% formalin for 15 min and immunostained with PAD_4_ antibody (1:100, Gene Tex Inc., CA, USA) overnight. The proteins were detected using Alexa-488 antibody (1:500, Molecular Probes, USA). Sections were observed and photographed with a Zeiss microscope (Carl Zeiss, Oberkochen, Germany).

Since the discovery of NETs in 2004 [3], there has been extensive research, but much of the specifics of NET formation remains elusive. NETs have a dual function of maintaining hematologic homeostasis and of defending the organism. They are released not only on exposure to pathogens such as bacteria, but also in sterile environments in autoimmune diseases such as lupus where they generate/trap autoantigens and aggravate inflammation. NETosis and NETs could play an important role in the initiation of autoimmune diseases. Different enzymes are involved in the formation and function of NETs. These include Myeloperoxidase, NADPH oxidase, Rac2 of the Rho family [5] and peptidyl arginine deiminase 4 (PAD4). PAD4 is a calcium dependent enzyme localized in the nucleus of neutrophils and is involved in the citrullination of histones [6–8]. NETs have been identified in kidneys and skin of lupus patients [9]. Studies show significant increase of PAD_4_ staining in brain of MRL/lpr mice compared to their congenic MRL/MPJ controls (Figure 1). Increase in PAD_4_ leads to increases in citrullination resulting in heightened generation of autoantigens and deamination of proteins such as antithrombin and histones H_2_A, H_3_ and H_4_ [10]. Pharmacological inhibition or deletion of PAD_4_ alleviates pathology in SLE. Further research is needed to establish definitive roles for the increased PAD_4_ expression in the lupus brain.

The use of electron microscopy and immunofluorescence has allowed the observation of pathogens binding to extracellular DNA released by NETosis. One study showed the capture and neutralization of circulating *Escherichia coli* when NETs were released into hepatic sinusoids, highlighting a benign role of NETs in quelling an infection [11]. In addition to microorganisms, NETosis can also be stimulated by proinflammatory cytokines (e.g., TNF-α, IL-8), platelets, activated endothelial cells (ECs), nitric oxide, monosodium urate crystals, and various autoantibodies [2]. While NETosis seems to be a benign host process intended to thwart infection, aberrant activation such as that in SLE can result in extensive self-damage.

Apoptosis or predetermined cell death occurs in different tissues in lupus [12–14]. NETosis is different from other forms of cell death such as apoptosis and necrosis with the nuclear as well as the granular membranes undergoing fragmentation. Apoptosis occurs through a family of proteins called caspases while NETosis occurs entirely independent of caspases and is not affected by the caspase inhibitor zVAD-fmk [15,16]. Although lupus is a sterile environment, neutrophils could be induced to release NETs by cell-to-cell crosstalk through soluble mediators or microvesicles released by activated endothelial cells or platelets [17,18]. Endothelial cells are activated in lupus while mean platelet number is inversely correlated with disease [19].

Neutrophils also interact with the adaptive immune system in complex ways many of which remains to be deciphered. NETs activate plasmacytoid dendritic cells (pDC) through TLR7 and TLR9 modulating inflammatory responses and prime T cells by reducing their activation threshold. NETs prime T cell responses rendering them sensitive to specific antigens and even to suboptimal stimuli [20–22]. One of the regulators of neutrophil homeostasis and migration is the IL-_23/17_ axis that regulates T cell differentiation [23,24]. The IL-_23_/IL-_17_ axis has a protective effect in human SLE. IL-17 has an inverse correlation with cardiac function and tumor frequency in some SLE cohorts, suggesting a protective effect possibly by neutrophil recruitment, inducing DC maturation, activation of macrophages, and NK cell and T-cell-induced cytolysis [25].

Another interesting feature of autoimmune diseases is the generation of antibodies against self-proteins. These antibodies along with immune complexes are deposited in tissues causing inflammation and functional aberrations. Immune complexes bind to NETs and are taken up by dendritic cells. Large quantities of NETs are present in the kidneys, skin, and blood of lupus patients, and their presence correlate with the disease activity [9,26]. We have data that show increased NETs in experimental lupus brain (Figure 1). Increased NETs in lupus could be because of increased NETs being released by the neutrophils or due to reduced elimination of the NETs. Once NETs are formed they gain access to the endosomal compartment of the B cells leading to generation of autoantibodies but the underlying mechanism that induces this phenomenon remains a mystery.

## Animal Models

One of the most widely used models is the MRL/*lpr* strain which contains *lpr* mutation in the *Fas* gene, a normal apoptotic regulator of lymphocytes. Loss of functional Fas causes lymphoproliferation, and mice have an accumulation of CD_4_^−^/CD_8_^−^ B_220_^+^ T-cells that are auto-reactive to dsDNA. Consequently, MRL/*lpr* mice develop a lupus-like phenotype from consequential robust immune complex formation. Important to note, however, while many of the manifestations of MRL/*lpr* mice mirror those of human SLE patients, MRL/*lpr* mice acquire massive lymphadenopathy which does not occur in humans [27]. NZBxNZW mice are another common strain generated from a cross between NZB and NZW mice (F_1_ hybrid), and are commonly used for genetic studies. While MRL/*lpr* mice do not have a sex bias for lupus traits, NZBxNZW have a female sex bias [27]. Mice can also have induced lupus phenotype due to injections of chemicals like pristane, an isoprenoid alkane, which induces Ab-complex formation [27,28]

## Autoantigens

NETosis provides a mechanism for stimulation of autoimmunity as the nuclear DNA, histones, and granule proteins released by the NETs serve as self-antigens. Hakkim et al. found that in a subset of patients with SLE, degradation of NETs was lessened compared to a pool of healthy, unrelated blood donors [29]. Failure to clear the autoantigens present in NETs allows for the prolonged exposure and development of autoimmunity to those antigens and subsequent pathology found in SLE.

Neutropenia or low neutrophil count is common in lupus patients due to its correlation with high levels of IFNα present in the body resulting in more NETosis. The bursting of neutrophils through the release of NETs enhances the development of SLE as the T and B cells become autoreactive and continuously produce autoantibodies [30].

In a normal host, DNase I degrades NETs. Interestingly, DNase I did not induce NET degradation in SLE patients except in a subset of SLE patients where MNase, a DNase from *Staphylococcus* caused NET degradation. As such, for some patients with SLE who are unable to degrade NETs, they may contain a deficient DNase I responsible for lack of NET clearance. In patients whose NETs are degraded by MNase, there appears to be a protective mechanism by the NETs resulting in resistance to clearance. Nonetheless, inability to degrade and clear NETs results in the exposure of the host immune system to antigens to which autoantibodies are formed – an association with SLE [29].

Plasmacytoid dendritic cells (pDCs) are stimulated by autoantibodies to induce the production of Type 1 IFNs causing an activation of both the innate and adaptive immune systems [31]. High levels of Type 1 IFN are associated with SLE, as the Type 1 IFN signature (IFNα) is present in SLE patients due to co-purification of low density neutrophils and mononuclear cells during gradient centrifugation [20]. In a study, Garcia-Romo et al. studied pediatric patients with SLE to determine whether anti-ribonucleoprotein (RNP) immunoglobulin (Ig) induced-NETosis would stimulate pDC activation. The pDCs of blood from healthy patients was compared to the supernatants of healthy or SLE neutrophil that were cultured with or without anti-RNP antibodies. The supernatants of anti-RNP IgG treated SLE neutrophils induced the greatest release of IFNα and activated pDCs leading to the production of Tumor Necrosis Factor-α (TNFα), cytokine alterations -interleukin-6 (IL-6), and interferon-inducible protein-10 (IP-_10_). Supernatants that were unable to form NETs were unable to activate pDCs. The results of the experiment concluded that SLE NETs are needed to activate pDCs and are excellent IFN inducers.

Further, one particular antimicrobial peptide, LL-_37_, has been identified to be extruded in NETs [32]. LL- _37_ induces inflammation via activation of pDCs, and is constitutively expressed in various immune cells, particularly neutrophils. LL-_37_ facilitates the recognition and uptake of extracellular DNA by pDCs. LL-_37_ has also been identified in the NETs of SLE, but not normal, patients. The result is the release of a type I IFN signature, particularly robust IFNα release, which in turn primes neutrophils for further NETosis as well as exacerbating the immune response [20].

## Mitochondria

NETosis has been categorized into multiple models of understanding. Reactive oxygen species are essential second messengers of suicidal NETosis as they promote the breakdown of the nuclear membrane into small vesicles allowing the decondensed chromatin to release DNA in the extracellular traps through cellular lysis [4]. The neutrophil loses all normal functioning. This burst of the plasma membrane and cell death have been compared to other pathogen-induced cell death processes such as apoptosis.

In vital NETosis the nuclear membrane remains intact despite releasing DNA through NET formation, and is able to continue normal neutrophil functioning. Stimuli received by toll-like receptors (TLR) leads to the decondensation of chromatin and vesicle formation altering the nuclear envelope for DNA release. Vital NETosis is of two types, one that does not rely on ROS activity while the other does. Vital NETosis that is ROS dependent results in the expulsion of mitochondrial DNA instead of nuclear DNA in NETs [4]. During suicidal NETosis, protein kinase C (PKC) activates the NADPH oxidase complex working with reactive oxidative species to promote the decondensation of chromatin through histones by enzyme peptidyl arginase deiminase 4 (PAD_4_), which citrullinates histones [33]. Notably, deficiency of PAD_4_ also impairs NET formation in the murine model [7], highlighting the necessity of PAD_4_ for occurrence of NETosis.

Previous research has found that immune complexes stimulate NETosis in an ROS dependent manner in mice with lupus-like disease. However, Lood et al., found that MLR/*lpr* lupus mice deficient in *Cybb* (a necessary component of NADPH), that is necessary for suicidal NETosis [34] had greater pathology. This implies that the pathology caused by NETosis in lupus occurs in a cellular-ROS-independent manner (i.e., cell viable NETosis). Instead, ROS generated by the mitochondria may be sufficient to provide a mechanism for NETosis. While pharmacological inhibition of ROS would ameliorate NETosis and possibly much of the pathology of SLE, the side effects of such inhibition would certainly be devastating to the host due to the putative resulting dysfunction of neutrophils. However, mitochondrial-ROS inhibitors could hold promise as specific therapeutics to ameliorate NETosis. Lood et al. show that upon prophylactic administration of MitoTEMPO, which selectively inhibits mitochondiral-ROS, MLR/*lpr* mice had attenuated lupus pathology with marked decreased in NETosis and IFNα release [34].

Finally, highlighting the importance of mitochondria in NETosis, Wang et al., highlight the importance of mitochondrial DNA (mtDNA) in cell-viable NETs. Here, mtDNA is more potent in NETs than nuclear dsDNA, and further provides another source of autoantibodies that elaborate SLE pathogenesis via IFNα production. These results are corroborated by the finding of anti-mtDNA antibodies in study of SLE patients [35]. Thus, mtDNA and anti-mtDNA antibodies can serve as a potential biomarker and therapeutic target respectively in lupus.

## Endothelial Dysfunction

Lupus increases the risk of endothelial dysfunction and premature cardiovascular disease. Low density granulocytes (LDGs), a proinflammatory neutrophil subset isolated from human lupus peripheral blood mononuclear cell (PBMC) fractions are deleterious to the endothelium, with potential implications in the development of premature atherosclerosis [36]. LDG NETs demonstrate increased externalization of MMP-_9_ compared to healthy control or to normal density SLE neutrophils. Immunofluorescence microscopy confirmed the presence of MMP-_9_ and MMP-_25_ in NETs which indicate that lupus LDGs distinctly upregulate specific MMPs in NETs and are primed for enhanced NETosis. Matrix metalloproteinases (MMPs) are present in neutrophil granules and get externalized during NETosis. In addition, immune complexes composed of MMP-_9_ and anti-MMP-_9_ in lupus induce NETosis. NETs that contain increased levels of MMP_9_ activates endothelial MMP-_2_ thereby altering the functional integrity of the endothelium. NETs from lupus LDGs induced increased accumulation of NET-derived MMP-_9_ in human umbilical vein endothelial cells (HUVECs) changing their contour, area, perimeter and shape.

Further, acetylcholine induced endothelium-dependent vasorelaxation was impaired in the thoracic aorta rings from C_57_BL/6 female mice in the presence of LDG-NETs, compared with control NETs. Therefore, studying the molecular mechanism by which NETs cause endothelial damage in SLE could lead to new therapeutic targets in treating SLE and its associated vascular complications.

## Granulopoiesis

Low density granulocytes (LDGs), which are highly granular, have previously been found to be unique in the blood of SLE patients following fractionation [37], which is presumed to be the response of potent IFNα [38]. These LDGs are atypical in the blood, have an abnormal phenotype, and have a hyperability to undergo NETosis. Whereas high density granulocytes require potent IFN signaling for NET formation, LDGs spontaneously form NETs [39]. This has been shown to result in both the damage of vascular endothelial cells, as well as further stimulation of pDCs to synthesize and release IFNα [9].

Further, in comparison to normal density granulocytes (NDGs) from both lupus and healthy patients, mitochondrial superoxide production was enhanced in lupus LDGs, which is critical for NET formation. Thus, NET formation in lupus LDGs occurs in a cellular-ROS independent, mitochondrial-ROS dependent manner.

## Lupus Glomerulonephritis

The most fatal manifestation of SLE is glomerulonephritis [40]. Glomerulonephritis is inflammation of the filtration apparatus of the kidneys, and patients typically present with hematuria and red blood cell casts in their urine. Progression of glomerulonephritis leads to kidney failure due to chronic insults to the glomerulus along with extensive fibrosis. Because of the inflammatory nature of glomerulonephritis, the pathology is usually a consequence of aberrant immune activation, as seen in SLE [41–43].

In patients with SLE, the most common form of glomerulonephritis is diffuse proliferative glomerulonephritis (DPGN) that results from anti-dsDNA-containing IgG immune complex deposition along with C3 accumulation, both of which result in inflammatory cell infiltration. NETosis has been implicated specifically in the pathogenesis of lupus nephritis. For example, NETs have been found in the kidneys of lupus patients [9]. Rother et al. found that SLE patients with nephritis or past cases of nephritis lack the capacity to degrade NETs. Consequently, these intact NETs are deposited in the glomerulus further stimulating an inflammatory response by exacerbating the immune system and inducing a Type I Interferon response [44].

Patients with SLE develop lupus nephritis because as the cells undergo a change, inducing vascular leakage through the intercellular junction protein, VE-cadherin. Mesenchymal cells are also produced from the β-catenin signaling from elastase associated with the high presence of NETs [45]. Serum samples of NET non-degraders had higher anti-dsDNA and antinuclear antibodies (ANA) titers than degraders [29]. The techniques of DNA staining and anti-myeloperoxidase (MPO) antibodies, allowed the colocalization and analysis of IgG deposition on NETs found in tubules and glomeruli in the kidneys of an SLE patient. The cohort allowed for the risk factors of impaired NET degradation and high levels of anti-dsDNA antibodies to be identified as a link to lupus nephritis [31]. A closer look into the pathology found a molecular interplay between glomerular endothelial cells and recruited neutrophils. Extensive damage to endothelial cells results in unique microparticle release containing Annexin V+ apoptotic chromatin. These microparticles enhance the ability of neutrophils to undergo NETosis, which aggravates the pathology. Importantly, in comparing plasma from SLE patients with normal healthy donors, these Annexin V+ microparticles were present only in SLE plasma [46].

## Neuropsychiatric Lupus

A significant amount of SLE patients suffer from central nervous system (CNS) complications including headaches, depression, psychosis, encephalopathy, coma, and more [47]. Despite research into such complications, much of the pathophysiology of what is deemed neuropsychiatric systemic lupus erythematosus (NPSLE) remains elusive. Thus far, it is known that compromising the integrity of the blood-brain barrier (BBB) in NPSLE likely leads to such neuropsychiatric symptoms [48,49] as a consequence of an inflammatory influx that results in brain injury [50]. Critical to the BBB is the surrounding endothelial cells that protect the CNS from peripheral circulation. Essentially, with the loss of the BBB, inflammatory cells and autoantibodies have access to the CNS and result in pathology of the brain [51].

While research points to an immune complex (IC) initiation of complement mediated apoptosis as a mechanism of the loss of BBB integrity, studies have also shown the detrimental effect of transmigration of neutrophils and the release of extracellular traps. In this model, IL-_1_β induces activation of ICAM-_1_ and VCAM-_1_ in mouse brain endothelial cells (MBEC), which results in transmigration of neutrophils. Following transmigration neutrophils change to a neurotoxic phenotype, initiating degranulation and NETosis, including the release of decondensed DNA associated with neurotoxic proteases, resulting in both neuronal cell death and BBB damage. Further, the NETosis occurs in a cell viable manner, meaning the mitochondrial DNA rather than cellular DNA is extruded, and the extrusion occurs in an active, rather than suicidal, manner [52].

With NETosis induced BBB loss of integrity, the brain can now also be exposed to auto-antibodies. Antibodies against *N*-methyl-*D*-aspartate (NMDA) receptors (ligand-gated calcium channels), specifically binding to the zinc binding site, have been implicated in SLE patients resulting in CNS symptoms resulting in loss of neuronal cell viability via a Ca^2+^ influx and subsequent apoptotic pathway [53]. As such, NETosis induced loss of BBB integrity can also result in further neurotoxicity through the introduction of NMDA autoantibodies [54].

## Other Organs

The manifestations of SLE of course extend beyond the brain and the kidneys. The integument is a prominent site of lupus pathology, and NETosis has been implicated here in this instance as well. IFNα released by pDCs results in an inflammatory influx at the dermal-epidermal junction and, via TLR_7_ and _9_, NETs provide stimulation to pDCs. The role of NETs in pDC activation does not appear to be necessary for the initial cutaneous insult, however, because depletion of neutrophils prior to cutaneous stimulation does not ameliorate pathology. Instead, NETs appear to be playing a chronic role in autoimmune stimulation [55]. Further research needs to be done on the role of NETosis in other manifestations of lupus, such as arthritis, pericarditis, pleuritis, and malar rash.

## Treatment

The current treatment of lupus includes a multitude of drugs aimed primarily at suppressing the overactive immune system while also targeting the particular symptoms and manifestations. In general, patients are treated with the anti-malarial drug chloroquine or hydrochloroquine. Patients are also given a cocktail of immunosuppressants, including steroids like prednisone, glucocorticoids, cyclophosphamide, rituximab, etc. [56,57]. In other words, there is a great variety of current regimens that may be catered to a particular patient; however, none are curative, and each has its own harmful side effects. The implication of NETosis in SLE is promising in that a potential specific therapeutic target (e.g., mitochondrial ROS, Annexin V+ microsomes) may be clinically useful.

The proteins NETs express or bear, depends on the stimuli and the environment [2]. In SLE, since they are generated even in the absence of microbial triggers and once generated aggravate the inflammatory symptoms, therapeutic intervention reducing or preventing NET formation could be beneficial. However, this would depend on whether the neutrophils will still remain viable and functional in case of infections, or else researchers would have to identify other upstream or downstream therapeutic targets.

A potential treatment includes the use of metformin, a drug canonically used to treat diabetes mellitus. Wang et al. already highlighted the presence of mtDNA auto- antibodies in SLE patients, thus identifying a potential therapeutic target. *In vitro*, metformin decreases mtDNA NET release, thus a clinic trial with metformin was conducted comparing SLE patients treated with conventional treatment to a metformin add-on group. In the study, metformin notably reduced lupus flares; however, NETosis and mtDNA were not directly measured in patients in this study [58]. Regardless, targeting the source of autoantibodies in NETosis and lupus, specifically mtDNA, appears to be clinically promising.

Additional to mtDNA, NETs have been found to have hypo-acylated histones on expelled DNA, which is both pro-inflammatory and pro-apoptotic [44,45,59]. Further, these free histones are preferentially recognized by auto- IgG in lupus [59]. Administration of a histone-deacetylase inhibitor (HDAC inhibitor) such as trichostatin A may hold promise in attenuating this response. For example, both Mishra et al. and Garcia et al. found that administration of HDAC inhibitors decrease the pathology in lupus nephritis in MRL/*lpr* mice [60,61]. However, at the time of the study the results may have been attributed to correction of epigenetics rather than NETosis, specifically.

Other components of the machinery behind NET release are potential targets. Inhibition of PAD_4_, which is necessary for histone citrullination and chromatin decondensation, negatively modulates NET formation [62]. Even more, inhibition in MLR/lpr mice also decreases the manifestations of lupus [63]. The caveat behind PAD_4_ inhibition, however, is the potential of infections by eliminating a potentially integral defense function of neutrophils [64]. Additionally, with the involvement of TLR_7_ and _9_ in chronic skin autoimmunity from lupus, inhibitors of the TLR_7_ and _9_ decreased cutaneous manifestations in NZB/W mice [55]. Such inhibitors hold therapeutic potential in humans for lupus-induced dermatitis.

Overall, the implication of NETosis in the pathogenesis of SLE is promising in identifying therapeutic targets beyond broad immunosuppression that lacks both specificity and efficacy. Further, targeting aberrant NETosis specifically, such as mtDNA targets, HDAC inhibitors, or mitochondrial ROS inhibitors can inhibit pathological NETosis while leaving benign NETosis intact, allowing what appears to be a normal mechanism of innate defense functional.
